# Epethelial Presence of *Trueperella pyogenes* Predicts Site-Level Presence of Cranial Abscess Disease in White-Tailed Deer (*Odocoileus virginianus*)

**DOI:** 10.1371/journal.pone.0120028

**Published:** 2015-03-24

**Authors:** Emily H. Belser, Bradley S. Cohen, Shamus P. Keeler, Charles H. Killmaster, John W. Bowers, Karl V. Miller

**Affiliations:** 1 Warnell School of Forestry and Natural Resources, University of Georgia, Athens, Georgia, United States of America; 2 Southeastern Cooperative Wildlife Disease Study, College of Veterinary Medicine, The University of Georgia, Athens, Georgia, United States of America; 3 Georgia Department of Natural Resources, Wildlife Division, Social Circle, Georgia, United States of America; INIAV, I.P.- National Institute of Agriculture and Veterinary Research, PORTUGAL

## Abstract

Cranial/intracranial abscess disease is an emerging source of significant mortality for male white-tailed deer (*Odocoileus virginianus*). Most cases of cranial/intracranial abscess disease are associated with infection by the opportunistic pathogen *Trueperella pyogenes* although the relationship between the prevalence of the bacteria and occurrence of disease is speculative. We examined 5,612 hunter-harvested deer from 29 sites across all physiographic provinces in Georgia for evidence of cranial abscess disease and sampled the forehead, lingual, and nasal surfaces from 692 deer. We used polymerase chain reaction (PCR) to determine presence of *T*. *pyogenes* from these samples. We found *T*. *pyogenes* prevalence at a site was a predictor for the occurrence of cranial abscess disease. Prevalence of *T*. *pyogenes* did not differ between samples from the nose or tongue although prevalence along the forehead was greater for males than females (p = 0.04), particularly at sites with high occurrence of this disease. Socio-sexual behaviors, bacterial prevalence, or physiological characteristics may predispose male deer to intracranial/cranial abscess disease. Determination of factors that affect *T*. *pyogenes* prevalence among sites may help explain the occurrence of this disease among populations.

## Introduction

Understanding disease epizootiology in wildlife populations is critical for characterizing population impacts, identifying at-risk segments of the population, and molding adaptive management efforts. This can lead to targeting hosts and regions predicted to be at risk of disease emergence and is critical as emerging wildlife-borne diseases continue to affect populations and biodiversity [1]. Cranial/intracranial abscess disease is a cause of morbidity and mortality in white-tailed deer (*Odocoileus virginianus*) across portions of the United States and Canada [2]. This disease disproportionately affects males ≥ 3.5 years old [2,3,4] and reports of recent significant mortality from this disease indicate it could have population level impacts [2,5]. The gender-related bias of intracranial abscess disease may be due to the unique behavioral patterns of adult males associated with sexual competition [3,4], or potential differences in the bacterial flora between male and female deer.

Cranial abscess disease results when pyogenic bacteria opportunistically invade cuts, abrasions, or injured pedicles, causing an infection of the cranial region [2,3]. Further penetration of the infection into the skull may occur along the suture between the parietal and frontal bones resulting in intracranial abscess disease, which is typically fatal [3]. Most abscesses are 1–3 cm in diameter with off-white, yellow or pale green viscous pus. An intense inflammatory reaction surrounds the infection. Clinical signs of deer with intracranial abscess disease include incoordination, lack of fear, blindness, weakness, depression, emaciation, circling, lameness, and fever [3].

Although numerous bacteria have been found associated with intracranial abscesses, *Trueperella* (*Arcanobacterium*) *pyogenes* [6] is most commonly isolated [2,3,4,5]. *T*. *pyogenes* is a Gram-positive, rod-shaped, nonmotile and non-spore forming bacterium with both proteolytic and hemolytic activities [7]. This opportunistic bacterium can be a resident bacteria along the mucosal surfaces of healthy cattle and swine, as well as other domestic species [7,8]. *T*. *pyogenes* is believed to be the most widely distributed and common opportunistic pathogen of mucosal surfaces in domestic animals [7,9]. *T*. *pyogenes* induces suppurative infections, in the form of abscesses, empyemas, and pyogranulomas [9]. *T*. *pyogenes* is also widespread in wild animals, as it has been associated with disease in blackbuck antelope (*Antilope cervicarpa*), bison (*Bison bonasus*), and camels (*Camelus dromedarius*). Although the prevalence of *T*. *pyogenes* in localized areas as it relates to white-tailed deer has been inspected [5,10], much of its association with deer in relation to cranial abscessation remains unknown. We examined *T*. *pyogenes* as a resident on white-tailed deer across a large landscape to determine if the prevalence of this bacterium is associated with the differential occurrence of this disease. In particular, our objectives were to determine if the prevalence of *T*. *pyogenes* on mucosal surfaces of white-tailed deer influenced the occurrence of cranial abscess disease at the site level. Then, because antagonistic behaviors differ across age and gender of white-tailed deer and these behaviors may influence the transmission of *T*. *pyogenes*, we sought to examine the influence of host specific variables (i.e., gender and age) on the occurrence of *T*. *pyogenes*. Lastly, to suggest if any age or gender related differences may explain site-to-site variation in the occurrence of cranial abscess disease, we determined if the occurrence of *T*. *pyogenes* on the mucosal layers of white-tailed deer differed across gender and ages at sites affected and unaffected by this disease.

## Materials and Methods

All animal care and experimentation in this research was approved by the University of Georgia Institutional Animal Care and Use Committee (AUP# A2011 01–004-Y3-A0). All permits required for this study were issued by the Georgia Department of Natural Resources under scientific collecting permit 29-WH-12–170. This study was conducted on 29 sites, 8 of which were private property. Permission to utilize animals harvested from public lands was granted by the Georgia Department of Natural Resources. Contact for future permissions on private lands should be made to the corresponding author.

**Fig 1:**
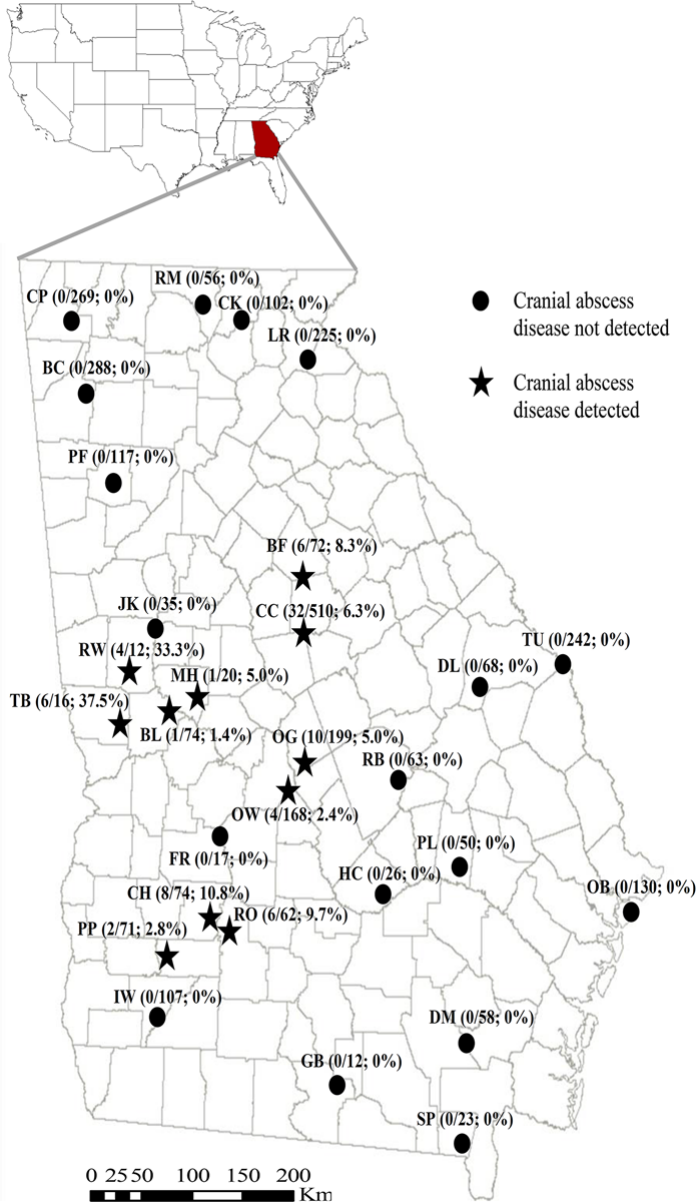
Location of 29 sites and the number of hunter-harvested adult (≥1.5 years old) male white-tailed deer testing positive for cranial abscess disease and the number examined for the presence of the cranial abscess disease in Georgia, USA during Fall 2011 and 2012.

From September 14, 2011 to January 15, 2012 and September 14, 2012 to January 15, 2013, we examined hunter-harvested deer from 29 sites across all physiographic provinces in Georgia, USA (Blue Ridge, Ridge and Valley, Piedmont, Upper Coastal Plain, and Lower Coastal Plain) for signs of cranial abscess disease. Concurrently, we sampled the forehead, lingual, and nasal linings from a randomly selected subset of deer at these sites to determine presence of *T*. *pyogenes*. Sites were selected to ensure a thorough spatial distribution across the state (Fig. 1). We recorded the age [11], gender, and location for each deer sampled.

Because cranial abscess disease is the direct predecessor of intracranial abscess disease and is grossly diagnosable without dissection, we used cranial abscesses as a measure of intracranial abscesses. Cranial abscess disease inspection was performed by a thorough examination of each deer’s cranium, with specific focus on the pedicles of bucks, and the comparable location on the cranium of does. Each pedicle and cranium was examined for necrosis of the skin, scabbing, or pus as previously described [3]. In addition, the skin around the base of the pedicle was palpated by hand to express pus, should a subcutaneous infection have been present. Any grossly visible indications of an infection on the forehead were classified as a cranial abscess.

### Bacterial Sample Collection and Processing

We sampled 692 harvested deer with a sterile, rayon-tipped applicator (Puritan Medical Products, Guilford, ME, USA) on the nasal mucosa, the lingual mucosa, and the forehead. We placed each sample in an individual 2.5 mL Cryosaver vial (Cryosaver, Hardy Diagnostics, Santa Maria, CA, USA) and froze them at -20°C until analyzed.

We extracted DNA from swabs using the DNeasy Blood and Tissue Kit (Qiagen, Valencia, CA, USA), per manufacturer’s instructions, with the following modifications: extra phosphate-buffered saline (PBS) (400 μl total) and lysis (AL) buffer (400 μl total), an additional five minutes to incubation for lysis, and an additional one minute to the final incubation step. These modifications to the DNA extraction protocol were done to compensate for the glycerol in the Cryosaver vials and were undertaken based on consultations with Qiagen technical support. We then stored extracted samples at -20°C until further processing.

We utilized polymerase chain reaction (PCR) in 25-μl reactions using GoTaq Flexi DNA Polymerase and GoTaq PCR reagents (Promega Corporation, Madison, WI, USA) per manufacturers recommendations. We targeted the *plo* gene, which is specific to *T*. *pyogenes* and has been reported in all strains, using primers F (5’-GGCCCGAATGTCACCGC) and R (5’-AACTCCGCCTCTAGCGC) [12]. We performed amplifications in an automated thermal cycler (BioRad DNA Engine, Hercules, CA, USA), with the following cycling conditions: 35 cycles, each consisting of a 1-min denaturation at 94°C, a 1-min annealing at 55°C, and a 1-min extension at 72°C; a final 5-min extension at 72°C. The PCR products were electrophoresed in ethidium bromide stained 1.5 (w/v) agarose gels. A 100 basepair DNA ladder (Promega Corporation) was used as DNA marker. The gel was visualized using an AlphaImager gel documentation system (ProteinSimple, Santa Clara, CA, USA) to determine presence or absence of the *plo* gene, and thus *T*. *pyogenes*. Precautions were taken to avoid contamination of samples, including separation of the areas for DNA extraction and PCR preparation, regular changing of gloves, and use of DNA-away (Molecular BioProducts, Inc., San Diego, CA, USA) for surface and equipment sterilization between batches. All PCR cycles included negative controls to ensure there was no contamination in the reaction mixture. We used *T*. *pyogenes* reference strains (JGS 189 and JGS 230) as positive controls for every cycle.

To ensure the reliability of our PCR process, a random subset of bands from 24 positive samples were extracted from the agarose gel using the QIAquick Gel Extraction Kit (Qiagen, Valencia, CA, USA) per manufacturer’s instructions and sent for sequencing to the Georgia Genomics Facility (University of Georgia, Athens, GA, USA). A Basic Local Alignment Search Tool (BLAST) search of the resulting sequences was used for identification [13]. Nineteen genetically sequenced samples were determined to be *T*. *pyogenes*. Five results were inconclusive.

### Data analysis

#### *T*. *pyogenes* prevalence and site-level occurrence of cranial abscess disease

We utilized a generalized linear model procedure with binomial errors fitted with a logit link to test if *T*. *pyogenes* prevalence on the different epidermal layers of deer affected the presence of cranial abscess disease among sites. The dependent variable was a binary measure, with cranial abscess disease presence or absence of the site classified as 1 or 0, respectively. Because of the sex-related bias of this disease [2,3,5], we considered sites in which an adult (≥ 1.5 years old) male deer was detected with a cranial abscess to be positive for this disease. Our focus was on site-to-site variability, but a priori we believed these populations to be distinct from each other because of the large distances separating them. Predictor variables included the prevalence (percentage) of *T*. *pyogenes* site on the head, nose, tongue, or overall (i.e., detection of *T*. *pyogenes* on any mucosal layer of deer) at each site. To examine for potential collinearity, Pearson correlations (r) were performed on all pairs of predictor variables. No variables that were significantly correlated (cutoff of r = ± 0.70) [14] were included in the same model. We used Akaike's Information Criteria (AIC) [15] with small sample adjustment (AICc) [16] to compare the relative goodness-of-fit for these eight models. We calculated ΔAIC_i_ values between the AIC value for the candidate model *i* and the lowest-ranked AIC value. The models with ΔAICc ≤ 2 were considered good candidates for explaining patterns in data, models with ΔAICc 2–7 had little support, and models with ΔAICc ≥ 10 had relatively no support. The data used in this analysis is available in S1 Dataset.

#### Host-specific factors associated with *T*. *pyogenes* detection

We conducted generalized linear mixed modeling (GLMM) analyses using the lme4 [17] and LMER Convenience Functions [18] to investigate host specific variables influencing *T*. *pyogenes* presence. The lme4 and LMER Convenience Functions are packages in Program R allowing flexible modeling structures in a maximum-likelihood framework. Our analyses used a logit link with binomial errors and were fitted using Laplace approximation. Our dependent variable was a binary measure, with *T*. *pyogenes* presence or absence at the head, nose, or tongue of each deer classified as 1 or 0, respectively. Our predictor variables were age and gender. Because most deer life history traits and disease susceptibility is driven by age and gender differences, we included a gender by age interaction term as a predictor variable as well. To examine for potential collinearity between age and gender, we performed Pearson correlation (r) between the two variables. To account for the unknown variation occurring at each site, we considered site a random variable. The data used in this and the subsequent analyses detailed below is available in S2 Dataset.

#### Host specific factors associated with *T*. *pyogenes* detection at unaffected and affected sites

We subsequently performed a secondary, separate set of generalized linear mixed models to investigate differences of *T*. *pyogenes* presence on the head for sites with cranial abscess disease and sites without cranial abscess disease using the same predictor, random variables, and binomial error structure as described above. We set our statistical significance value at p ≤ 0.05 for all analyses. All analyses for this study were conducted using the program R v.2.10.1 (R Development Core Team, 2009).

## Results

### *T*. *pyogenes* prevalence and site-level occurrence of cranial abscess disease

We examined 5,612 white-tailed deer of which 3,166 of which were adult males. We found no cranial abscesses on any of the 2,446 female deer and male deer <1 year old examined. We detected cranial abscesses in 80 (2.5%) of adult male deer examined (see [4] for more details on age and gender related risks of this disease). Of the 29 sites surveyed, 11 (37.9%) met the threshold criteria to be considered as having cranial abscess disease present at the site level for our analysis (Fig. 1).

We sampled 692 deer for *T*. *pyogenes*, 449 were males; 196 were females (Table 1). Three sites did not provide any gender or age data. Thus, gender data was not available for 47 deer and age data was unavailable for 162 deer. Each deer was sampled on the head, nose, and tongue, for a total of 2,070 samples analyzed. We detected *T*. *pyogenes* at each site, albeit at varying prevalence. Overall prevalence of *T*. *pyogenes* among sites from hunter-harvested deer ranged from 16.7% to 100% (Table 1). No collinearity amongst detection on the different mucosal layers was identified (Table 2). The best approximating AIC model (*w*_i_ = 0.25; Table 3) indicated that overall prevalence of *T*. *pyogenes* was the most important factor predicting presence or absence of cranial abscess disease at a site. The second best approximating model (*w*_i_ = 0.23), which was not statistically different from the “best” model, indicated that nasal detection of *T*. *pyogenes* was the most important factor predicting presence or absence of cranial abscess disease on a site. Prevalence of *T*. *pyogenes* at both the nose and forehead was also a good candidate (*w*_i_ = 0.15), as was prevalence at just the forehead (*w*_i_ = 0.12). None of the other models were able to predict a site’s vulnerability to cranial abscess disease.

**Table 1:** Prevalence and the number of hunter-harvested white-tailed deer sampled (M = Male; F = Female; U = Unknown) for *Trueperella pyogenes* across 29 locations in Georgia during 2011 and 2012. Prevalence is reported as the percent of samples that were positive on the head, nose, tongue, and overall (i.e., detection of *T*. *pyogenes* on any surface) ± 95% confidence intervals.

Location by county (and code)	*N*			Head (%)			Nose (%)			Tongue (%)			Overall (%)		
	M	F	U	Lower	Mean	Upper	Lower	Mean	Upper	Lower	Mean	Upper	Lower	Mean	Upper
Baker (IW)	8	20	2	4.4	13.3	31.6	26.0	43.3	62.3	8.4	20.0	39.1	33.1	50.0	66.8
Burke (DL)	19	11	-	23.2	40.0	59.2	37.7	56.7	74.0	2.6	10.0	27.7	57.3	76.7	89.4
Chatham (OB)	22	8	-	8.4	20.0	39.1	2.6	10.0	27.7	4.4	13.3	31.6	26.0	43.3	62.3
Dougherty (CH)	28	2	-	8.4	20.0	39.1	1.2	6.7	23.5	1.2	6.7	23.5	13.0	26.7	46.2
Fannin (CK)	16	14	-	8.4	20.0	39.1	10.6	23.3	42.7	4.4	13.3	31.6	26.0	43.3	62.3
Floyd (BC)	25	5	-	2.6	10.0	27.7	1.2	6.7	23.5	1.2	6.7	23.5	6.3	16.7	35.5
Gilmer (RM)	8	5	-	15.1	38.4	67.7	6.2	23.1	54.0	2.7	15.4	46.3	32.3	61.5	84.9
Habersham (LR)	19	6	-	38.9	60.0	78.2	5.3	16.0	36.9	5.3	16.0	36.9	46.4	68.0	84.3
Harris (TB)	10	10	5	38.9	60.0	78.2	63.1	84.0	94.7	35.3	56.0	75.0	72.5	92.0	98.6
Houston (OW)	25	-	-	5.3	16.0	36.9	31.8	52.0	71.7	5.3	16.0	36.9	42.6	64.0	81.3
Laurens (RB)	16	2	-	7.4	22.2	48.1	10.7	27.8	53.6	7.4	22.2	48.1	36.1	61.1	81.7
Lee (PP)	3	-	27	26.0	43.3	62.3	43.9	63.3	79.5	34.6	53.3	71.2	76.5	93.3	98.8
Lowndes (GB)	4	1	-	1.1	20.0	70.1	1.1	20.0	70.1	1.1	20.0	70.1	17.0	60.0	92.7
Meriwether 1 (JK)	14	7	-	9.1	23.8	47.5	1.7	9.5	31.8	0.2	4.8	25.9	9.1	23.8	47.5
Meriwether 2 (RW)	11	3	-	56.1	85.7	97.5	42.0	71.4	90.4	9.6	28.6	58.0	73.2	100.0	100.0
Paulding (PF)	23	2	-	1.4	8.0	27.5	5.3	16.0	36.9	7.6	20.0	41.3	15.7	32.0	53.6
Putnam 1 (BF)	24	6	-	10.6	23.3	42.7	13.0	26.7	46.2	4.4	13.3	31.6	34.6	53.3	71.2
Putnam 2 (CC)	30	-	-	33.1	50.0	66.8	6.3	16.7	35.5	0.2	3.3	19.1	40.8	60.0	76.8
Screven (TU)	12	13	-	10.2	24.0	45.5	1.4	8.0	27.5	12.9	28.0	49.6	21.8	40.0	61.1
Sumter (FR)	12	12	-	0.0	0.0	17.2	31.4	50.0	68.6	3.3	12.5	33.5	36.9	58.3	77.2
Talbot (BL)	12	13	-	0.2	4.0	22.3	28.3	48.0	68.2	5.3	16.0	36.9	31.8	52.0	71.7
Telfair (HC)	11	2	-	0.4	7.7	37.9	15.1	38.5	67.7	0.0	0.0	28.3	15.1	38.5	67.7
Toombs (PL)	4	16	9	16.0	31.0	51.0	36.0	55.2	73.0	13.4	27.6	47.5	52.5	72.4	86.6
Twiggs (OG)	30	-	-	20.5	36.7	56.1	1.2	6.7	23.5	1.2	6.7	23.5	26.0	43.3	62.3
Upson (MH)	3	3	2	0.7	12.5	53.3	21.5	50.0	78.5	10.2	37.5	74.1	25.9	62.5	89.8
Walker (CP)	24	5	1	4.4	13.3	31.6	0.0	0.0	14.1	6.3	16.7	35.5	13.0	26.7	46.2
Ware 1 (SP)	15	9	1	0.2	4.0	22.3	3.2	12.0	32.3	1.4	8.0	27.5	7.6	20.0	41.3
Ware 2 (DM)	9	3	-	11.3	33.3	64.6	6.7	25.0	57.2	6.7	25.0	57.2	35.4	66.7	88.7
Worth (RO)	12	18	0	26.0	43.3	62.3	34.6	53.3	71.2	13.0	26.7	46.2	60.9	80.0	91.6

**Table 2:** Pearson correlations (r) for fixed effect variables used in our modeling exercises. First, we examined if the detection of *Trueperella pyogenes* on one mucosal layer was associated with higher chance of detecting it at other mucosal layers. White-tailed deer were sampled along the forehead, nose, and tongue for presence of *T*. *pyogenes*. The prevalence of *T*. *pyogenes* on these mucosal layers across deer sampled at each site was used as predictor variables in subsequent modeling exercises to determine if associations with increased risk of cranial abscess disease at the site level. We then ensured no collinearity between the age and gender. No variables were significantly correlated enough to be excluded from being in the same model (cutoff of r = ± 0.70).

	r	df	t value	Pr(>|t|)
Head*Tongue	0.39	27	2.18	0.04
Head*Nose	0.38	27	2.11	0.04
Nose*Tongue	0.63	27	4.22	<0.01
Age*Gender	-0.17	525	-3.85	<0.01

**Table 3:** Akaike information criteria with small sample bias adjustment (AICc); number of parameters (K), ΔAICc, Akaike weights (*w*) for candidate models (*i*) relating prevalence of detecting *Trueperella pyogenes* on the mucosal surfaces of deer to the occurrence of cranial abscess disease at 29 sites across Georgia, USA in 2011–2012. The detection of *T*. *pyogenes* on any mucosal surface (overall detection) has the lowest AICc score.

Model structure and description^a^	K^b^	AICc	ΔAICc^c^	*w*_i_ ^d^
O	2	37.69	0.00	0.25
N	2	37.80	0.11	0.23
N + H	3	38.61	0.92	0.15
H	2	39.16	1.47	0.12
T	2	40.01	2.32	0.08
N + T	3	40.13	2.44	0.07
H + T	3	40.48	2.79	0.06
N + H + T	4	41.30	3.61	0.04

^a^ Models correspond to prevalence of *T*. *pyogenes* on: O = overall (i.e., detection at any of the three dermal linings sampled), H = Forehead, N = Nasal, T = Tongue.

^b^Number of estimating parameters in approximating model.

^c^ Models with ΔAICc ≤ 2 were considered good candidates for explaining patterns in field data, models with ΔAICc 2–7 had little support, and models with ΔAICc > 10 had relatively no support.

^d^Akaike weight.

### Host-specific factors associated with *T*. *pyogenes* detection

There was no significant collinearity between age and gender in our sample (Table 2). Because age and/or gender were not available for 3 sites (DL, PL, PP), only 26 sites were included in this analysis. Based on the 527 animals of known age and gender, we found no evidence that age or gender had an effect on the probability of *T*. *pyogenes* detection overall, in the nose, or on the tongue (Table 4). There was a greater probability of detecting *T*. *pyogenes* on the head of male deer (p = 0.05; Table 4).

**Table 4:** Parameter estimates (logit scale) for a model of the risk of an individual white-tailed deer testing positive for *Trueperella pyogenes* on A) overall (detection at any of the three dermal linings sampled), B) head, C) nose, and D) tongue. Standard errors (SE), z values, and probabilities that a coefficient differs from 0 are also presented. Age and gender data was available for 527 deer across 26 sites. Residual degrees of freedom was 522 for all models.

	Covariate	Estimate	Coefficient (SE)	z value	Pr(>|z|)
Overall	Intercept	-0.61	0.42	-0.15	0.89
	Age	-0.56	0.11	-0.53	0.60
	Gender	-0.32	0.49	-0.67	0.51
	Age*Gender	0.21	0.14	1.48	0.14
	Site^1^	0.57	-	-	-
Head	Intercept	-2.23	0.57	-3.88	<0.01
	Age	0.12	0.13	0.88	0.38
	Gender	1.20	0.63	1.91	0.05
	Age*Gender	-0.19	0.17	-1.12	0.26
	Site^1^	1.25	-	-	-
Nose	Intercept	-0.70	0.45	-1.55	0.12
	Age	-0.09	0.11	-0.77	0.43
	Gender	-1.39	0.55	-2.52	0.01
	Age*Gender	0.37	0.16	2.33	0.02
	Site^1^	0.76	-	-	-
Tongue	Intercept	-1.98	0.56	3.51	<0.01
	Age	-0.03	0.16	0.20	0.84
	Gender	-0.11	0.67	0.16	0.87
	Age*Gender	0.16	0.19	0.83	0.41
	Site^1^	0.06	-	-	-

^1^ Site was considered a random effect in the model. Thus, it is a variance estimate.

### Host specific factors associated with *T*. *pyogenes* detection at unaffected and affected sites

Based on the 309 deer on sites without cranial abscesses and where age/gender data was available (n = 16), neither age (p = 0.54), gender (p = 0.18), nor their interaction (p = 0.17) affected the probability of *T*. *pyogenes* detection on the head. However, from the 218 deer on sites where abscesses were observed and age/gender data was available (n = 10), there was a greater probability of *T*. *pyogenes* occurring on the head of males (p = 0.04; Table 5). Age was not significant (p = 0.49), nor was the age and gender interaction (p = 0.31).

**Table 5:** Parameter estimates (logit scale) for a model of the risk of an individual white-tailed deer testing positive for *Trueperella pyogenes* on the head for sites with cranial abscess disease (CAD) and sites without cranial abscess disease. Standard errors (SE), z values, and probabilities that a coefficient differs from 0 are also presented. 218 deer of known age and gender from 10 sites with cranial abscesses and 309 deer from 16 sites without cranial abscesses were included in the respective models. Residual degrees of freedom was 213 for the “with CAD” model and 304 for the “without CAD” model.

	Covariate	Estimate	Coefficient (SE)	z value	Pr(>|z|)
With CAD	Intercept	-2.53	1.04	-2.44	0.01
	Age	0.16	0.23	0.69	0.49
	Gender	2.43	1.17	2.07	0.04
	Age*Gender	-0.28	0.28	-1.01	0.31
	Site^1^	1.53	-	-	-
Without CAD	Intercept	-2.19	0.68	-3.22	<0.01
	Age	0.10	0.17	0.62	0.54
	Gender	1.04	0.78	1.34	0.18
	Age*Gender	-0.33	0.24	-1.37	0.17
	Site^1^	0.90	-	-	-

^1^ Site was considered a random effect in the model. Thus, it is a variance estimate.

## Discussion

Although intracranial abscess disease is a natural mortality factor in white-tailed deer populations [2,3,5], few studies have assessed the prevalence of the purported etiological agent in a deer herd [5,10]. Our results show *T*. *pyogenes* is a common resident bacterium of white-tailed deer in Georgia, USA. Although prevalence of *T*. *pyogenes* varied dramatically among sites, no site had a prevalence of zero. In contrast, Turner et al. [10] did not detect *T*. *pyogenes* on white-tailed deer in 3 of 6 physiographic provinces of Maryland, USA. However, comparison between studies is limited because only nasal and head swabs were used in the Maryland study, and bacterial isolation and identification techniques were different from our study.

Although cranial/intracranial abscess disease disproportionately affects mature bucks ≥3.5 years [2,3], we observed no effect of gender or age on the prevalence of *T*. *pyogenes* on the nose, tongue, or overall. However, males were more likely to have *T*. *pyogenes* on the forehead on sites where cranial abscess disease was detected, suggesting that behavioral or physiological characteristics of males may lead to an increased likelihood of *T*. *pyogenes* presence. Antler rubbing on trees or sparring between males and associated social grooming [19] may facilitate bacterial transmission. Antler rubbing and sparring also may result in injuries and abrasions [20], providing opportunities for infection. Further, these antagonistic behaviors may agitate an active infection and hasten disease progression. While these behaviors may explain why males are more likely to have cranial abscess disease, they cannot explain why there was no effect of age or gender on *T*. *pyogenes* prevalence for sites without abscesses. Rather, differences among sites in prevalence of *T*. *pyogenes* and the incidence of cranial abscess disease may be related to differences in the virulence factors aiding epithelial attachment and subsequent colonization of *T*. *pyogenes* among various sites.

Although not statistically demonstrated, it seems there is a higher prevalence of *T*. *pyogenes* in the western portion of the Piedmont and Upper Coastal Plain physiographic provinces of Georgia. Thus, environmental factors may play a role in the prevalence of *T*. *pyogenes* and, consequently, cranial/intracranial abscess. For example, Baumann et al. [2] reported no skull lesions characteristic of abscesses on 299 skulls from Texas. Furthermore, nasopharyngeal and antler-base swabs of nine Maryland bucks (≥2.5 year-old) were positive for *T*. *pyogenes* [5], but no swabs were positive from any of ten healthy adult (≥2.5 year-old) male white-tailed deer in south Texas, which suggests that the bacteria may be less prevalent in arid climates. Because our results indicate a strong relationship in geographic distribution between prevalence of *T*. *pyogenes* and cranial abscess disease, studies assessing factors that may affect *T*. *pyogenes* prevalence, cranial/intracranial abscess disease incidence are prudent.

## Supporting Information

S1 Dataset Site prevalence of *T*. *pyogenes*. (CSV)

S2 Dataset Age, gender, and mucosal layer occurrence of *T*. *pyogenes*. (CSV)

## References

[1] Grogan LF, Berger L, Rose K, Grillo V, Cashins SD, Skerratt LF. Surveillance for emerging biodiversity diseases of wildlife. PLOS Pathog. 2014;10: 5 Available: e1004015. doi: 10.1371/journal.ppat.1004015 10.1371/journal.ppat.1004015 PMC4038591 24875394

[2] Baumann CD, Davidson WR, Roscoe DE, Beheler-Amass K. Intracranial abscessation in white-tailed deer of North America. J Wild Dis. 2001;37: 661–670. 10.7589/0090-3558-37.4.661 11763729

[3] Davidson WR, Nettles VF, Hayes LE, Howerth EW, Couvillion CE. Epidemiologic features of an intracranial abscessation/suppurative meningoencephalitis. J Wildl Dis. 1990;26: 460–467. 2250322 10.7589/0090-3558-26.4.460

[4] Cohen BS, Belser EH, Killmaster CH, Bowers JW, Irwin BJ, Yabsley MJ, et al Epizootiology of cranial abscess disease in white-tailed deer. J Wildl Dis. In press. 10.7589/2014-05-129 25984774

[5] Karns GR, Lancia RA, DePerno CS, Conner MC, Stoskopf MK. Intracranial abscessation as a natural mortality factor for adult male white-tailed deer (*Odocoileus virginianus*) in Kent County, Maryland, USA. J Wildl Dis. 2009;45: 196–200. 19204349 10.7589/0090-3558-45.1.196

[6] Yassin AF, Hupfer H, Siering C, Schumann P. Comparative chemotaxonomic and phylogenetic studies on the genus *Arcanobacterium* Collins *et al*. 1982 emend. Lehnen *et al*. 2006: proposal for *Trueperella* gen. nov. and emended description for the genus *Arcanobacterium* . Int J Syst Evol Micr. 2011;61: 1265–1274. doi: 10.1099/ijs.0.020032-0 20622055 10.1099/ijs.0.020032-0

[7] Jost HB, Billington SJ. *Arcanobacterium pyogenes*: molecular pathogenesis of an animal opportunist. Antonie van Leeuwenhock. 2005;88: 87–102. 16096685 10.1007/s10482-005-2316-5

[8] Madsen M, Sorensen GH, Aalbaek B, Hansen JW, Bjorn H. Summer mastitis in heifers: Studies on the seasonal occurrence of *Actinomyces pyogenes*, *Peptostrepcoccus indolicus* and *Bacteroidaceae* in clinically healthy cattle in Denmark. Vet Microbiol. 1992;30: 243–255. 1348381 10.1016/0378-1135(92)90118-d

[9] Moore R, Miyoshi A, Pacheco LGC, Seyffert N, Azevedo V. *Corynebacterium* and *Arcanobacterium* In: Gyles CL, Prescott JF, Songer JG, Theon CO, editors. Pathogenesis of bacterial infections in animals. 4th ed Ames: Wiley-Blackwell; 2010 pp. 113–147.

[10] Turner MM, DePerno CS, Conner MC, Eyler TB, Lancia RA, Klaver RW, et al Habitat, wildlife, and one health: *Arcanobacterium pyogenes* in Maryland and Upper Eastern Shore white-tailed deer populations. Infect Ecol Epidemiol. 2013;3: 10. Available: 3402/iee.v3i0.19175. Accessed 20 January 2014. 10.3402/iee.v3i0.19175 PMC3737440 23930157

[11] Severinghaus CW. Tooth development and wear as criteria of age in white-tailed deer. J Wildl Manage. 1949;13: 195–215.

[12] Billington SJ, Jost BH, Cuevas WA, Bright KR, Songer JG. The *Arcanobacterium (Actinomyces*) *Pyogenes* hemolysin, pyolysin, is a novel member of the thiol-activated cytolysin family. J Bacteriol. 1997;179: 6100–6106. 9324258 10.1128/jb.179.19.6100-6106.1997 PMC179514

[13] Hall TA. BioEdit: a user-friendly biological sequence alignment editor and analysis program for Windows 95/98/NT. Nucl Acids Sympos Ser. 1999;41: 95–98.

[14] Dormann CF, Elith J, Bacher S, Buchmann C, Carl G, Carré G, et al Collinearity: a review of methods to deal with it and a simulation study evaluating their performance. Ecography. 2012;36: 27–46.

[15] Akaike H. Information theory and an extension of the maximum likelihood principle In: Petrov BN, Csaki F, editors. Second International Symposium on Information Theory, Budapest: Akademia Kiado; 1973 pp. 267–281.

[16] Hurvich CM, Tsai CL. Regression and time series model selection in small samples. Biometrika. 1989;76: 297–307.

[17] Bates DM, Maechler, Bolker BM. lme4:Linear mixed-effects models using S4 classes version 0.999375–39. 2011. Available: http://CRAN.R-project.org/package5lme4. Accessed 20 May 2013.

[18] Tremblay A (2011) LMERConvenienceFunctions: A suite of functions to back-fit fixed effects and forward-fit random effects, as well as other miscellaneous functions, Version 1.6.7. 2011. Available: http://CRAN.R-project.org/package5LMERConvenienceFunctions. Accessed 20 May 2013.

[19] Forand KJ, Marchinton RL. Patterns of social grooming in adult white-tailed deer. Am Midl Nat. 1989;122: 357–364.

[20] Marchinton LR, Hirth DH. Behavior In: Halls LK, editor. White-tailed deer ecology and management. Washington, DC: Wildlife Management Institute; 1984 pp. 129–168.

